# Small Biological Fighters Against Cancer: Viruses, Bacteria, Archaea, Fungi, Protozoa, and Microalgae

**DOI:** 10.3390/biomedicines13030665

**Published:** 2025-03-08

**Authors:** Pathea Shawnae Bruno, Peter Biggers, Niyogushima Nuru, Nicholas Versaci, Miruna Ioana Chirila, Costel C. Darie, Anca-Narcisa Neagu

**Affiliations:** 1 Biochemistry & Proteomics Laboratories, Department of Chemistry and Biochemistry, Clarkson University, Potsdam, NY 13699-5810, USA; brunop@clarkson.edu (P.S.B.); biggerpd@clarkson.edu (P.B.); nurun@clarkson.edu (N.N.); versacna@clarkson.edu (N.V.); 2 Laboratory of Animal Histology, Faculty of Biology, “Alexandru Ioan Cuza” University of Iași, Carol I Bvd. 20A, 700505 Iasi, Romania; miruna.chirila@student.uaic.ro; 3 Faculty of Medicine, “Grigore T. Popa” University of Medicine and Pharmacy, University Street No. 16, 700115 Iasi, Romania

**Keywords:** cancer, viruses, bacteria, archaea, fungi, protozoa, microalgae, biomedicine

## Abstract

Despite the progress made in oncological theranostics, cancer remains a global health problem and a leading cause of death worldwide. Multidrug and radiation therapy resistance is an important challenge in cancer treatment. To overcome this great concern in clinical practice, conventional therapies are more and more used in combination with modern approaches to improve the quality of patients’ lives. In this review, we emphasize how small biological entities, such as viruses, bacteria, archaea, fungi, protozoans, and microalgae, as well as their related structural compounds and toxins/metabolites/bioactive molecules, can prevent and suppress cancer or regulate malignant initiation, progression, metastasis, and responses to different therapies. All these small biological fighters are free-living or parasitic in nature and, furthermore, viruses, bacteria, archaea, fungi, and protozoans are components of human and animal microbiomes. Recently, polymorphic microbiomes have been recognized as a new emerging hallmark of cancer. Fortunately, there is no limit to the development of novel approaches in cancer biomedicine. Thus, viral vector-based cancer therapies based on genetically engineered viruses, bacteriotherapy, mycotherapy based on anti-cancer fungal bioactive compounds, use of protozoan parasite-derived proteins, nanoarchaeosomes, and microalgae-based microrobots have been more and more used in oncology, promoting biomimetic approaches and biology-inspired strategies to maximize cancer diagnostic and therapy efficiency, leading to an improved patients’ quality of life.

## 1. Introduction

The origin of malignancy is not yet well understood and cancer remains a leading cause of death [1]. Genomic instability and accumulation of genetic mutations, infections and inflammation, inappropriate diet and nutrition, environmental exposure to harmful xenobiotics, intake of toxins, and exposure to stress have been recognized as the most important risk factors contributing to carcinogenesis and cancer development [2,3,4,5]. Certain viruses [6,7], bacteria [8,9,10], archaea [11,12], fungi [13,14], protozoa [15,16], microalgae [17,18], and their related structural components/bioactive compounds/metabolites/toxins have been associated with the etiology, pathogenesis, development, and/or personalized theranostic of animal and human cancers, due to their oncogenic, oncolytic/tumor suppressor, or circumstantial/dual role in various cancer types or subtypes and among different patients. Therapeutic microbes can exert a dual role in cancer therapy, so pros and cons must be deeply debated before using them alone and in combination with traditional anti-cancer therapies [9]. Engineered viral vector-based cancer therapies could become a standard approach in the therapy of majority types of cancer, so numerous cancer vaccines employing viral-based strategies have been developed to induce anti-tumor responses [7,19]. Viral vector-based approaches also help for the attenuation of various degrees of pain among cancer patients due to modulation of pain cascade [20]. Bacteriotherapy is a novel anti-cancer approach, alone or in combinatorial mode with conventional therapies, and can lead to tumor regression and increased survival rate [10]. Archaeal metabolites might influence the tumor microenvironment and carcinogenesis [12]. Various protozoa exert anti-tumor mechanisms, so it is necessary to exploit their potential for clinical applications [15]. Last but not least, bioactive compound from microalgae can exert anti-cancer activity and could be used for developing new drugs [17].

Interactions between cancer cells and microbial cells are crucial in carcinogenesis and the progression of tumors [21]. Polymorphic microbiomes have been recently recognized as a new emerging hallmark of cancer, because microbes can be directly carcinogenic, impact host immune responses to promote cancer development, and modulate the effects of conventional anti-cancer therapies [22,23]. The term microbiome is actually used to define a complicated microbial ecosystem, consisting of microbiota, which includes bacteria, viruses, archaea, fungi, and protozoa, as well as their environment, consisting of structural components and signaling molecules, such as microbial proteins/peptides, lipids, polysaccharides and nucleic acids, metabolites/toxins and other molecules, as well as other specific conditions in their milieu [23,24,25]. The most studied in association with cancer is the gut microbiome, but other microbiomes from different tissues and organs, such as the skin, lungs, genito-urinary system, and breasts, as well as the intratumoral microbiome, emphasized an increased interest in cancer research [23]. Dysregulated microbiota can create a microenvironment which supports uncontrolled cell growth by chronic infection that leads to persistent inflammation, allowing for reactive oxygen species (ROS) generation and promotion of DNA damage and failure in DNA repair mechanisms, also associated with damage to proteins and lipids [26]. Thus, microbiome-induced oxidative stress (OS) is involved in cancer development, activating a variety of transcription factors (TFs) that control the expression of inflammatory cytokines and chemokines as well as other molecular factors involved in altered cell growth, mutation and genetic instability, and inhibition of apoptosis [26].

In total, 15–20% of all human cancers worldwide are associated with bacterial, viral, and parasitic infections [6,27,28], and of all cancer cases, 10–15% are caused by several well-known viruses [29]. However, certain commensal viruses can protect against cancer [30]. Tumor cells are sensitive to oncolytic viruses (OVs), which can directly invade and destroy them, or are able to enhance anti-tumor immune responses in the tumor microenvironment (TME) [31,32]. Certain bacteria are also associated with human cancers [33]. More and more evidence suggests that archaea are also associated with cancer and secrete different bioactive compounds that can inhibit cancer cell growth [11,12]. Fungi also influence carcinogenesis, modulating host immunity and producing bioactive metabolites [13]. Numerous species of protozoa and their components exhibited anti-cancer potential that resulted in a novel research direction for development of effective cancer therapies [15]. It is also known that numerous microbial metabolites have a dual role in cancer progression [12]. Last but not least, green microalgae could be delivered to hypoxic tumor regions to increase in situ oxygenation and emphasize significant anti-cancer activities, especially due to the improvement of the therapeutic effects of oxygen-consuming therapies such as radiotherapy and photodynamic therapy (PDT) [18,34,35].

Several therapeutic approaches are currently available for cancer, but endoscopy, surgery, chemotherapy, radiotherapy, targeted therapy, and immunotherapy, often in combination, are the primary options, but not for all patients [36]. It was well recognized that clinicians use pharmaceuticals as the first tool to fight against diseases, including cancer [37]. Unfortunately, chemotherapy is still characterized by poor absorption of anti-cancer drugs into tumor tissues, undesirable side effects on healthy cells, and development of resistance to chemotherapy [11]. In total, 50% of all cancer patients receive radiotherapy during their illness, but tumors can also develop resistance [36]. PDT is a non-invasive cancer treatment strategy able to kill tumor cells based on cytotoxic reactive oxygen species (ROS) that are produced by excitation of a photosensitizer under laser irradiation in the presence of oxygen [38]. In addition, sonodynamic therapy (SDT), a minimally/non-invasive anti-cancer therapy that involves chemical sonosensitizers and high-intensity focused ultrasound (HIFU), is able to destroy or denature target malignant tissues, or amplify the drug’s ability to transfer into tumor cells or TME to decrease tumor growth potential, induce apoptosis, and enhance immune response [39].

Fortunately, there is no limit to the development of novel approaches in cancer biomedicine that allow for the crossroad between traditional oncomedicine and novel tools of holistic medicine that can help to exploit biological resources to improve patients’ life quality [40]. Thus, over the past decades, gene therapy has become an important strategy for cancer treatment [41], so oncolytic viruses emerged as promising therapeutic agents for anti-cancer treatment and several OVs are now approved for virotherapy in different human cancers [42]. Furthermore, viral systems are able to enhance the efficacy of chemotherapy [43]. Recently, bacteria were recognized as promising organisms for cancer therapy [44]. Thus, magnetotactic bacteria (MTB) can be considered as prospective agents for cancer treatment, due to their flagellar-based swimming ability as well as the magnetosome chain’s ability to guide them towards cancer cells under magnetic fields [45]. Fungi interactions with the bacterial microbiome and the host tissues enabled them to be a target for cancer theranostics [13]. Protozoa can also combat cancer [15]. Microalgae-inspired microrobots (AIMs) can be used for optimizing the beneficial effects of traditional anti-cancer therapies or to minimize their destructive side effects [46]. Finally, nanotheranostics that employ biomimetic approaches based on biology-inspired strategies could maximize cancer diagnostic and therapy efficiency, leading to improved patients’ quality of life.

## 2. Viruses

Viruses can influence oncogenic pathways, promoting cancer development, or infect and kill cancer cells, being designed as potential anti-cancer agents. Furthermore, engineered viral vectors and viral vector-based cancer treatments, which offer an ideal combination between efficient tumor suppressor gene delivery and stimulation of host immune system for an appropriate anti-tumor response, represent promising approaches able to revolutionize the oncology [7]. As long as 10–15% of the worldwide cancers that occur annually are virus-induced [6,29], identification of oncogenic DNA and RNA viruses, understanding their molecular mechanisms of action leading to tumorigenesis and cancer development, as well as discovery of new approaches for treatment and prevention of viral infections known to lead to cancer are crucial.

### 2.1. Pro-Cancer

Bacteria, fungi, protozoa, and viruses are involved in the building and functioning of microbiome from the skin, oral cavity, lungs, genito-urinary tract, and gut based on the reciprocal relationship between microbiota and human cells and tissues [47,48]. Almost 15–20% of all human cancers worldwide are caused by microbial infections including bacteria, parasites, and oncogenic viruses/oncoviruses (OVs) [6,27,28], 10–15% of human cancers worldwide being caused by seven viruses, including the Epstein–Barr virus (EBV), hepatitis virus B (HBV), hepatitis virus C (HCV), human T-lymphotrophic virus-1 (HTLV-1), human papillomavirus (HPV), Kaposi’s sarcoma herpesvirus (KSHV)/human herpes virus 8 (HHV8), and Merkel cell polyomavirus (MCPyV) [29]. Viruses are obligatory intracellular parasites, so many viral proteins are able to reprogram host cellular pathways involved in proliferation, differentiation, apoptosis, and other types of cell death, genomic integrity and mutation, and immune surveillance [49]. OVs integrate their viral genes into the host genome, activate viral oncogenes, and promote oncogenic proteins, thus inducing malignant transformation and cancer development through disruption of cell cycle regulation, apoptosis, and DNA damage repair (DDR) mechanisms, leading to uncontrolled cell proliferation [28]. Genomic instability is one of the most important factors by which OVs promote cancer development [50].

The morphological and functional properties of the skin layers as well as the skin homeostasis are maintained by commensal microbe regulation [51]. Commensal viruses constitute approximately 40% of the skin microbiome, in which double-strand DNA lytic or temperate bacteriophages are dominant, accounting for 90% of the skin virome, whereas the viruses that infect eukaryotic cells account for 10% [51]. HPVs are the most important skin-infecting virus responsible for cutaneous squamous cell cancers, followed by MCPyV for Merkel cell carcinoma, and human KSHV for Kaposi’s sarcoma [52]. HPV preferentially infects undifferentiated proliferative basal cells of epithelia, which are capable of dividing [53]. There is evidences suggesting that HPV, MCPyV, and EBV can also have a potential role for lung cancer [29]. In small ruminants, the Jaagsiekte sheep retrovirus (JSRV) and enzootic nasal tumor virus (ENTV) are able to promote oncogenic transformation of differentiated epithelial cells in the lung and nasal cavities, respectively, leading to cancer, such as lepidic pulmonary adenocarcinoma analogous to human lung adenocarcinoma, via their envelope glycoproteins, which are able to dysregulate several signaling pathways involved in cell proliferation [54]. HPV-associated viruses, including human immunodeficiency virus (HIV), HSV, human polyomavirus 1 (BKV), and human polyomavirus 2 (JCV) are responsible for the occurrence and progression of bladder cancer [55]. Approximately 99.7% of cervical cancer cases are caused by genital high-risk HPV infection, and HPV/HSV-2 co-infection can result in a higher risk of developing cervical cancer [56,57].

The human gastrointestinal tract and related organs, including liver, gallbladder, and pancreas, contain bacteriophages and other viruses that infect both prokaryotic cells, including bacteria and archaea, and eukaryotic cells, participating in tissue homeostasis and health maintenance [58]. Thus, HPV is responsible for oral, esophageal, colorectal, anal cancers, EBV for oral, esophageal, gastric, hepatic, and colorectal cancer, and HIV for anal cancer [58]. HPV has been also proposed as a risk factor for oral squamous cell carcinoma, the most common type of malignancy of the oral cavity, as well as EBV and herpes simplex virus type 1 (HSV-1), which have been also proposed as OVs involved in oral cavity carcinogenesis [53]. HBV, HCV, and HDV infection is one of the major risk factors for hepatocellular carcinoma (HCC) development [59]. Thus, people infected with HBV early in their lifetime can experience oncogenic transformation of hepatocytes through viral DNA integration, genetic dysregulation, chromosomal translocations, chronic inflammation, and oncogenic pathways regulated by viral protein expression that lead to hepatocarcinogenesis many years later [60].

Several viruses with oncogenic potential that act alone or in combination are involved in breast cancer (BC), such as high-risk HPV and EBV that also cause human cervical and oral/nasopharyngeal carcinoma, mouse mammary tumor virus (MMTV) which causes BC in mice, and bovine leukemia virus (BLV) which causes cancers in cattle [61]. Other viruses are also implicated in the oncogenic transformation of cells and cancer development [47]. For example, West Nile virus, a single-stranded RNA flavivirus with a high tropism for the central nervous system, can contribute to the development of aggressive brain tumors, including glioblastoma multiforme (GBM) and dysembryoplastic neuroepithelial tumors [62]. Cancer-causing animal viruses are diverse, including retroviruses such as feline leukemia virus (FELV), an enveloped RNA virus that causes immune suppression, lymphoma, leukemia, and fibrosarcomas in domestic and some wild *Felidae* worldwide [63], bovine leukemia (BLV), Rous sarcoma, avian leukosis, and herpesviruses [64].

### 2.2. Anti-Cancer

Haddad et al. (2021) showed that viruses used for tumor treatment have been divided into three categories: (i) wild-type of genetically engineered oncolytic viruses, such as herpes simplex virus (HSV) or adenovirus, that selectively invade and divide into malignant cells and destroy them before releasing progeny viruses; (ii) replication-deficient viruses, such as retrovirus or adenovirus, which are used as targeted gene-delivery systems, thus assuring the introduction of anti-oncogenes or tumor suppressor genes in cancer cells, stimulating their differentiation, inducing apoptosis, or decreasing proliferation, and (iii) replicating non-lytic viruses, such as retrovirus, which selectively divide in malignant cells while releasing progeny viruses in a non-lytic manner, the therapeutic effect resulting from the genes they deliver [65]. Moreover, suicide gene therapy is based on heterologous expression of viral specific enzymes that convert, together with the cancer cell’s own enzymatic machinery, the non-toxic form of a chemotherapeutic drug into the cytotoxic form, leading to lysis of transgene-expressing cells and those surrounding them [66]. Immunomodulatory vectors also stimulate expression of strong antigens on the tumor cell surface or production of factors that attract immune cells [66]. Several oncolytic vectors, such as herpesvirus, adenovirus, reovirus, poxvirus, and Newcastle disease virus, are able to replicate only within breast cancer cells and spare normal cells [43]. Targeted viral gene therapy and use of viral vectors to deliver local immunotherapy emphasized numerous progressions and challenges in numerous types of cancer, including breast cancer (BC) [43,67,68], colorectal cancer (CRC) [69,70], glioblastoma (GBM) [65,66,71], pancreatic ductal adenocarcinoma (PDAC) [72,73], ovarian cancer [74,75], head and neck cancer (HNC) [19], melanoma [76], osteosarcoma [77], prostate cancer [78], hepatocellular carcinoma (HCC) [79,80], bladder cancer [81], and others.

Both commensal and oncolytic viruses express the good that viruses do. As mentioned above, commensal viruses constitute approximately 40% of the skin microbiome, in which bacteriophages are dominant [51]. T cell immunity against commensal HPV suppresses skin cancer in immunocompetent hosts [30]. The commensal gut virome comprises predominantly bacteriophages, but also contains eukaryotic DNA and RNA viruses such as adenovirus, astrovirus, rotavirus, bocavirus, picornavirus, anellovirus, and picobinavirus [82]. Commensal gut viruses play an important role in colon cancer suppression by maintenance of intestinal intraepithelial lymphocytes through a type I interferon-independent manner [83]. Commensals also play an important role in preventing respiratory and invasive disease through inhibition of colonization and expansion of potential pathogens, immune system modulation, and stimulation of barrier function [84]. The asymptomatic presence of respiratory viruses, including adenovirus and thinovirus, is commonly found in the nasopharynx [84].

Viral vector-based anti-cancer treatments are based on viral vectors that act as gene delivery devices, cancer vaccines, and targeted oncolytic therapeutics [7]. Thus, oncolytic virotherapy uses oncolytic viruses or genetically engineered/recombinant viruses to selectively infect tumor cells, followed by their replication inside them and the destruction of malignant cells, associated with significant boosting of the host’s immune response [85]. Oncolytic viruses emerged as promising therapeutic agents for anti-cancer treatment over the past decades and could become a standard tool in cancer therapy that is able to revolutionize the oncological domain [7,42]. The molecular biology of viruses as well as virus–host cell interaction or virus–bacteria interaction led to the opportunity to use viruses to treat malignant tumors [31]. Point mutations and viral recombination are the main mechanisms that lead to genetic change of viruses [86]. Oncolytic viruses can be used to genetically modify cells and trigger the expression of tumor-specific antigens, or kill tumor cells, releasing soluble antigens and interferons, and finally, to boost the anti-cancer immune responses [87,88]. For example, human enteroviruses are an appropriate source for obtaining oncolytic enterovirus variants due to their low or lack of pathogenicity and since they can be attenuated for reduction in safety risks and to enhance their tropism [31].

Viral vector-based gene therapy is based on local or systemic administration of viral vectors and the delivery of therapeutic exogenous/foreign genes into the target malignant cells or within TME, using oncolytic retroviruses, adenoviruses (OAd), herpes simplex virus (oHSV), or adeno-associated viruses (AAVs), which can be used for cancer treatment [41,89]. Viral vector systems are engineered by removing pathogenic genes from their genomes and inserting therapeutic genes able to produce anti-tumor immune factors or cytotoxic proteins to kill malignant cells [41]. The principal OV therapy advantage over chemotherapy results from virus’ ability of self-propagation, allowing them to replicate and infect more tumor cells until all mass of tumor cells are eradicated [90]. Additionally, viruses infect efficiently a wide range of cancer cells, expressing multiple genes, and also emphasizing immunogenicity [91].

Lv et al. (2023) used a modified AAV as a gene delivery vector that can selectively target BC cells, offering a new therapeutic agent for the treatment of EpCAM-positive BC and other tumor types [67]. OAds become promising options for BC, especially for invasive diseases [92]. Thus, OAds encoding the human sodium/iodide symporter (hNIS), a transmembrane protein that can transport iodide ions across cell membrane, have been used for targeting of breast cancer stem cells (BCSC)-enriched estrogen receptor-positive (ER+) paclitaxel-resistant [92] or pancreatic cancer cells [93]. OAds are able to deliver transgenes specifically to cancer cells while sparing normal cells, making them take up radioactive iodine, which can be exploited for noninvasive imaging and radiotherapy [92]. oHSV, a double-stranded DNA virus that belongs to the *Herpesviride* family can be also genetically engineered by adding or replacing genes through genetic recombination to target cancer cells while sparing normal cells, killing them mainly by activating the host innate or/and adaptive immune responses [94]. Thus, talimogene laherparevec (T-VEC) is an HSV-1-derived OV variant that was approved for clinical use by the United States Food and Drug Administration (FDA) [90]. Recombinant mumps virus, a single-stranded negative-sense RNA genome that belongs to the family of Paramyxoviridae, has a potential to act as an oncolytic agent that could be a promising cancer therapeutic agent, emphasizing anti-cancer activity against various cancers, including advanced gynaecological cancer [95]. Variola virus, the source of the smallpox vaccine, is a brick-shaped, double-strand DVA virus of the genus Orthopoxvirus of the family Poxviridae, which has been used as an oncolytic agent that selectively kills cancer cells, and is a delivery vehicle for anti-cancer transgenes as well as a vaccine carrier for tumor-associated antigens and immunoregulatory molecules in cancer immunotherapy [91]. Human endogenous retroviruses (HERs) are genes derived from ancestral exogenous retroviruses integrated in our germline DNA, which seem to play divergent roles in carcinogenesis, including BC [96].

Additionally, oncolytic virotherapy becomes a potential anti-cancer therapy based on viral NPs [97]. Plant viruses and plant virus-based nanoparticles (PVNPs) can be engineered for targeted therapeutic agent delivery, imaging applications, and immunotherapy [98]. Thus, PVNPs emphasize high biocompatibility, biodegradability and safety for the environment [99], stability, and adjustable surface functionality, resulting in promising nanotechnologies able to fight against cancer [100]. First of all, PVNPs are used as nanocarriers for drug delivery in cancer therapy [101]. PVNPs exhibit different shapes and functionalities. Thus, the rod-shape tobacco mosaic virus (TMV) particle/nanotube is 300 nm long and 18 nm in diameter, with a 4 nm hollow channel, and possesses a capsid made of 2130 identical copies of coat protein (CP) helicoidally arranged [102]. TMV has been used as a delivery platform for pheanthriplatin (PhenPt-TMV), a DNA-binding platinum (II) anti-cancer drug candidate, which can be released in acidic tumor microenvironments (TME) [103]. PhenPt-TMV efficacy has been demonstrated using a mouse model of triple-negative breast cancer (TNBC) [103]. Furthermore, TMV can be also loaded with mitoxantrone (MTO), a topoisomerase II inhibitor used to treat various cancer types, resulting in a _MTO_TMV platform with efficacy in a panel of cancer cell lines and a mouse model of TNBC [104]. Moreover, functionalized TMV coat protein monomers and oligomers were tested as nanocarriers for anti-cancer peptides that target the transmembrane as well as the extracellular domain of the neuropilin 1 (NRP1) receptor in cancer cells, disrupting NRP1 interactions and downregulating the AKT downstream survival signal or NRP1-dependent angiogenesis [102]. The plant potato virus X (PVX) elongated filaments are 515 nm long and 13 nm in diameter and emphasize good tumor penetration properties [101]. Thus, PVX NP can be loaded with doxorubicin (PVX-DOX) with efficacy in a panel of cancer cell lines for ovarian, breast, and cervical cancer, the treatment resulting in reduced tumor growth [101]. The affinity of PVX towards malignant B cells in non-Hodgkin’s B cell lymphomas (NHL) was also demonstrated [105]. Thus, the PVX nanocarrier enables a good delivery of monomethyl auristatin to human B lymphoma cells in a NHL mouse model, with inhibitory effects on lymphoma growth and improved survival [105]. Cowpea mosaic virus (CPMV) NPs have strong immunostimulatory properties that can reshape the immunosuppressive TME in the murine orthotopic ovarian cancer model by modulating cytokine secretion [106]. Several anti-tumorigenic roles of engineered viruses are summarized in Table 1.

## 3. Bacteria

As well as viruses, bacteria are important components of human microbiomes, classified into gut, oral, respiratory, vaginal, and skin microbiomes that develop a dynamic symbiosis with the host’s cells and tissues [107]. The number of bacteria in the human body was estimated to be 3.8 × 10^13^, whereas the number of human cells was estimated at 3.0 × 10^13^ [108]. The total mass of bacteria in the body is low, being estimated to be almost 0.2 kg [108]. Additionally, bacteria are important residents in human tumors, inhabiting both cancer cells and immune cells, whereas the bacterial composition varies according to tumor type [109].

### 3.1. Anti-Cancer

Key probiotic bacteria are considered as a promising tool in cancer treatment due to their systemic immunomodulating properties [26]. Thus, probiotics can act as antigenotoxic, anti-tumorigenic, and antioxidative agents that stimulate the innate defense mechanisms of hosts, preventing cancer onset and development [26]. Microbiota manipulation, mainly intestinal or vaginal microflora, becomes a potential tool in preventing carcinogenesis and cancer progression in colorectal cancer [110], prostate cancer [111], and breast cancer [112], or cervical cancer, respectively [113]. Fecal microbiota transplantation (FMT) from heathy donors into patients’ intestines contributes to the remodeling of microbiota characteristics, boosting host immune checkpoint inhibitors performance by regulating tumor-immune cell interaction and altering microbial metabolites [114]. Thus, FMT can affect how metastatic melanoma patients respond to anti-programmed cell death protein 1 (PD-1) immunotherapy [115]. Application of vaginal microbiota transplantation (VMT) to restore a healthy microflora contributes to the treatment of cervical cancer by stimulating the local immune response, leading to better elimination of HPV [116], and might be also applied in the management of ovarian cancer therapy [117]. Oral microbiota transplantation (OMT) can be used to fight against common radiotherapy-induced complications among nasal, oral, and laryngeal cancer patients [118]. Thus, bifidobacteria are Gram-positive anaerobes from the normal human microflora, known for certain anti-cancer actions, resulting from their anti-proliferative, pro-apoptotic, and antioxidant properties [119,120]. *Lactobacillus* and *Bifidobacterium* strains interact with proteins involved in cell cycle regulation, inhibiting proliferation of malignant cells, and activating pro-caspases and pro-apoptotic BAX proteins, while downregulating the anti-apoptotic B-cell lymphoma 2 (BCL-2) protein [120]. In the gut microbiome from patients with non-small-cell lung cancer, *Bifidobacterium bifidum* was abundant in patients responsive to anti-cancer therapy [121]. Evidence also suggests a deep association between gut microbiota and BC progression [122]. Furthermore, in the presence of a breast tumor, local microbiota change compared to normal tissue [122]. Thus, specific bacterial species modulating cellular pathways involved in cell proliferation and apoptosis have been identified within breast tumors [122]. Moreover, BC subtypes characterized by higher oxidative stress (OS) are enriched in bacteria that produce mycothiol able to detoxify ROS [109].

Genetically modified bacteria are designed to express reporter genes, cytotoxic proteins or anti-cancer agents, and tumor-specific antigens, triggering a specific immune response in patients, whereas bacterial toxins and enzymes selectively cause apoptosis and cell cycle inhibition and could be combined with anti-tumor drugs or radiation therapy to enhance the efficacy or to alleviate the side effects of conventional anti-cancer therapies [10]. Additionally, bacterial spores can be also exploited as delivery agents for cytotoxic/therapeutic proteins/peptides or as vectors for gene therapy [10].

Bioengineered bacteria for cancer immunotherapy have the ability to target and to penetrate tumors, exert motility and chemotaxis, induce inflammatory response, and evade the immune system, but induce anti-tumor immunity [123]. These bacteria can be loaded with therapeutic drugs [123]. Thus, *Salmonella typhimurium*, a Gram-negative bacillus that causes local and systemic infections in humans and animals, is a facultative anaerobe of Enterobacteriaceae, which aggregates and proliferates inside TME more than any other bacteria, stimulating inflammation and promoting anti-tumor immunity [124,125]. Modified and less toxic attenuated *S. typhimurium* can be used as a platform for anti-tumor DDSs in cancer treatment, inhibiting tumor growth and metastasis or promoting apoptosis in ovarian murine tumors [124]. Moreover, *S. typhimurium* strains deliver toxins that induce apoptosis in cancer cells [126]. Proteomics-based methods contributed to characterization of bacterial toxins, such as bacteriocins, favoring the development of anti-cancer drugs based on these bacterial toxins [127]. Bacteriocins, such as nisin from *Lactococcus lactis*, enterocin from *Enterococcus* sp., plantaricin from *Lactobacillus plantarum*, pediocin from *Pediococcus acidilactici*, bovicin from *Streptococcus bovis*, microcins from *Klebsiella pneumonie*, and others from *Lactococcus garvieae*, *Bacillus amyloliquefaciens*, and *Lacobacillus delbrueckiiare*, were able to distinguish between cancer and heathy cells and induce apoptosis in various cancer cell lines [127]. Bacteria can also fight against cancer cells by depletion of the nutrients and oxygen required for cancer cell metabolism [127]. For developing new treatment options in brain metastasis from BC, *Clostridium perfringens* enterotoxin (CPE) in interaction with claudin-4, used as a possible biomarker for BC, can induce disruption of cancer cell membrane permeability and an influx of calcium ions, followed by cancer cell death [128]. Moreover, the C-terminal of CPE conjugated to NPs able to cross the brain–blood barrier could act as DDS to treat the brain metastases of BC [128]. *Pseudomonas aeruginosa* exotoxin A-based immunotoxins (PE-ITs) components are very poisonous for all cells but can be changed to damage only cancer cells in targeted cancer therapeutic delivery and to reduce cancer cell resistance [129]. Anthracyclines are glycoside antibiotics, including daunorubicin and doxorubicin (DOX) produced by *Streptomyces peucetius* var. *caesius* actinobacterium, as well as their derivatives, largely used for their potent anti-cancer activities in acute leukemia and various types of solid tumors, due to their ability to trigger both DNA double-strand breaks and histone eviction [130].

Chen et al. (2020) showed that the delivery of bacteria able to target tumor hypoxic regions, i.e., *Bifidobacterium longum*, followed by that of biotinylated lipid NPs coated with perfluorohexane (PFH/BL-NPs), can enhance the imaging of solid tumors and improve the efficiency of high-intensity focused ultrasound (HIFU) treatment in solid tumors by enhancing the ability of NPs to target solid tumors as well as increasing their retention time and the effects of the engineered HIFU synergists [131]. Moreover, non-pathogenic strains of *Escherichia coli* also possess the ability to selectively target, colonize, and proliferate within the solid tumors, especially in the regions characterized by low oxygen [132]. Thus, Zeng et al. (2024) developed the CGB@ICG, an engineered *E. coli* strain genetically incorporating acoustic reporter proteins and thermo-inducible cytolysin A (CLYA), a bacterial protein capable of forming pores in the tumor cell membrane that inhibits tumor progression, and chemically modified with indocyanine green on the bacterial surface [132]. For tumor regression, Wu et al. (2023) reported that probiotic facultative anaerobe *E. coli* Nissl 1917 (EcN) functionalized and formed self-propelled microrobots (EcN-Dox-Au) for doxorubicin delivery and photosensitizer gold nanorods for targeting, penetration, and accumulation into deep hypoxic regions of tumors [133].

Image-guided and thermally controlled bacteria could serve as imaging agents and delivery platforms for anti-tumor treatment [132]. Thus, bacteria-mediated tumor therapy showed promising potential for advanced cancer therapy based on the ability of the bacteria to act as delivery vectors, resulting in bacteria-driven drug delivery systems serving as promising carriers able to enhance drug penetration in solid tumors [44,132,133]. However, most commonly used therapeutic bacteria can exhibit pathogenicity and insufficient efficacy in the TME [134]. Magnetotactic bacteria (MTB) are motile Gram-negative aquatic microorganisms that synthesize and contain magnetosomes, intracellular NPs of magnetic iron oxide or iron sulfide minerals, which both have putative applications for targeted cancer therapy, including localized drug delivery, tumor monitoring, and magnetic hyperthermia [45]. MTBs can be guided by external magnetic fields, and are driven towards and deeply penetrate the hypoxic area in tumors, emerging as intelligent drug carriers [45]. MTB strains, such as AMB-1, are used for enhancing photothermal therapy (PTT) that engages thermal ablation of tumors [135]. The non-pathogenic natural purple photosynthetic bacteria (PPSB) can be also considered for cancer immunotheranostics, using bio-optical window I and II near-infrared (NIR) light [134]. Several anti-cancer activities and applications of bacteriotherapy are summarized in Table 2.

### 3.2. Pro-Cancer

Bacteria are recognized as opportunistic tumor inhabitants, producing environmental stress that alters the tumor microenvironment (TME) [136]. However, only several species of bacteria are recognized as oncobacteria directly related with carcinogenesis. Thus, *Escherichia coli* preferentially colonizes cancerous lesions in colorectal cancer (CRC) and sustains bladder cancer cell line progression through epithelial-to-mesenchymal transition (EMT), overexpression of CD44, NANOG, SOX2, and OCT4 stemness biomarkers, and metabolic reprogramming, demonstrated by ROS and metabolic biomarkers [136,137,138]. Over 97% of the gut microbiome consists of bacteria in the colon and plays a dual role in the pathogenesis of cancer [23]. It was estimated that 50% of the global population carries *Helicobacter pylori*, but only a small population of all infected individuals develops gastric cancer, resulting in approximately 5% of cancers caused by *Helicobacter pylori* infection [5,6,139]. *H. pylori* infection is significantly associated with gastric cancer [140], as well as gastric lymphoma, including marginal zone B-cell lymphoma of the mucosa-associated lymphoid tissue and diffuse large B-cell lymphoma [141]. To emphasize the positive association between *H. pylori* infection and gastric cancer risk, isolated *H. pylori* strains were classified as high virulent/type I, intermediate, and reduced virulent/type II strains, depending on the expression of the virulence factor cytotoxin-associated gene (c*agA*), the corresponding protein (CagA), and the vacuolating cytotoxin (VacA) [142]. The molecular mechanisms underlying *H. pylori* infection in gastric tumorigenesis are mainly based on dysregulation of STAT3, NF-kB, Hippo, and Wnt/β-catenin signaling pathways, *H. pylori* infection-inducing oxidative stress (OS), DNA damage, chronic inflammation, deregulation of cell apoptosis, and TME alterations [139]. *Mycobacterium tuberculosis* and *Mycobacterium avium* are implicated in lung cancer, whereas *Mycobacterium ulcerans* was correlated with skin carcinogenesis [143]. *Fusobacterium nucleatum*, a human oral Gram-negative anaerobic mycobiont associated with periodontal disease development, is involved in the progression of various tumors, such as colorectal, pancreatic, esophageal, and BC, through enhancement of cell proliferation, establishment of a tumor-promoting immune environment, and the induction of a resistance to chemotherapy that leads to poor disease outcome [144].

Various species of the genus *Staphylococcus* have been associated with tumorigenesis and tumor progression in many cancers, such as BC, skin cancer, bladder cancer, colon cancer, oral cancer, lung cancer, glioblastoma, and lymphoma [145]. *Staphylococcus aureus*, a common commensal Gram-positive bacterium that colonizes the human skin and nasopharynx in many individuals, produces toxins, exo-enzymes, and adhesion molecules that may induce malignant cellular changes, so chronic infections with *S. aureus* can promote tumor growth in skin and oral cancers [146]. Inflammation, DNA damage and downregulation of DNA repair, disruption of cellular signaling pathways, and generation of an immunosuppressive microenvironment are several factors that contribute to cancer progression in *S. aureus* infection [146]. A high bacterial load of *S. aureus* was associated with actinic keratosis, as a skin premalignant lesion, as well as cutaneous squamous cell carcinoma, common in severely photodamaged skin [147]. Bacterial infection, as a risk factor associated with cancer, can generate reactive oxygen species (ROS) in lesion-associated cells [148]. Taking into account that elevated levels of ROS are a hallmark of cancer [149], transcriptomics- and proteomics-based approaches demonstrated that secretomes of several skin lesion-associated clinical isolates of *S. aureus* strains can induce the overexpression of biomarkers associated with increased oxidative stress and downregulation of DNA repair mechanisms in human keratinocytes [147]. *Streptococcus gallolyticus* ssp. *gallolyticus*, a Gram-positive asymptomatic bacterium known as an opportunistic species of the human gut microbiota, produces tannase that degrades tannic acid (TA), an important component of human diet with anti-malignant proprieties, decreasing or silencing anti-cancer properties of TA [150]. Intestinal microbiota dysbiosis promotes local inflammation and development of gastrointestinal cancers [119,151]. There are studies that support an association between enterotoxigenic *Bacteroides fragilis* toxin and colon cancer formation in experimental models [152,153]. It is known that *B. fragilis* produces biofilm for colonization in the intestinal tract, leading to pro-carcinogenic inflammatory reactions, and produces this zinc-dependent toxin, which is a metalloprotease that targets epithelial tight junctions, leading to E-cadherin cleavage, resulting in enhanced NF-kB and WNT/β-catenin [152]. This toxin induces activation of signal transducers and activators of transcription 3 (STAT3) and increases the IL17 level that promotes epithelial cancer cell survival and proliferation [153,154].

The human respiratory tract is populated by a diverse community of commensals and potential pathogens, including pneumococcus (*Streptococcus pneumoniae*), *Haemophilus influenze*, *Moraxella catarrhalis*, and *Staphylococcus aureus*, which can turn occasionally into pathogens [84]. The mucosal layer in the lung is colonized by a diverse commensal bacterial community originating in the gastrointestinal tract, which provokes inflammation associated with lung adenocarcinoma (LUAD) development, induced by *KRAS* mutation and tumor suppressor p53 loss, by activating lung-resident T cells that produce IL-17 and other molecules which promote inflammation and tumor cell proliferation, angiogenesis, tissue remodeling, or metastasis [151].

## 4. Archaea

Archaea are common single-cell microorganisms with no nucleus or defined organelles present in animal and human microbiomes, mainly from the gut and oral cavity [12,155,156]. In the gastrointestinal tract, archaea account for up to 4% of all microorganisms and no archaeal pathogens have yet been identified [155]. However, archaea act as a double-edged sword, secreting different bioactive compounds that can inhibit cancer cell proliferation [11], or producing oncogenic metabolites that might influence the TME and promote carcinogenesis by microbiota modulation, aberrant signaling pathways, ROS production, DNA damage, inflammation, genotoxicity, tumor cell proliferation, and cell differentiation [12]. Archaeosomes are a new generation of stable polar lipid-based carriers similar to liposomes, synthetized from membrane natural lipids extracted from archaea or synthetic archaeal lipids that can transport and deliver anti-cancer-loaded drugs, proteins, peptides, genes, antioxidants, and cells to the tumor site [156,157]. Thus, archaeosomes induce tumor-protective CD8^+^ CTL responses and facilitate innate immunity by promoting natural killer cells and dendritic cells’ infiltration into the tumor site [157]. Consequently, archaeosomes are important vehicles in vaccine delivery, stimulating antigen-specific, humoral, and cell-mediated immune responses [156]. It was reported that the synthetized nano-archaeosomes containing paclitaxel emphasized a higher cytotoxic effect compared to free-form paclitaxel on breast cancer (BC) cell lines, such as MDA-MB-231 [158,159]. Recently, thermostable nano-archaeosomes, composed of archaeal lipids derived from hyperthermophilic archaeon *Aeropyrum pernix* K1 loaded with the anti-cancer drug doxorubicin, have been reported as a next-generation drug carrier for BC treatment due to their cytotoxic effect on the MCF7 BC cell line based on apoptosis and cell cycle arrest at the G0/G1 phase [160].

## 5. Fungal Infections

Human mycobiome consists of more than 400 species of fungal microorganisms mainly belonging to three phyla (*Ascomycota*, *Basidiomycota*, and *Chytridiomycota*) that inhabit different body sites [13,161]. Fungi are common inhabitants of a healthy digestive tract [161], accounting for only 0.1% of the total gut microbes, so gut mycobiome represents a small but crucial component of the gut microbiome, in close synergistic and regulatory association with bacteria [162]. There are multiple forms of fungus, such as yeast and hyphae [161]. The human gut mycobiota are dominated by yeast, including *Saccharomyces*, *Mallasezia*, and *Candida* genera [163]. *C. albicans*, a gut commensal and opportunistic pathogen, typically exists as yeast, but yeast and hyphae co-occur in the gastrointestinal tract, this morphogenetic switch controlling the dynamic balance between commensalism and pathogenic behavior [164]. Other fungi, such as *Aspergillus* and *Cladosporium* can be also introduced in GI by food [161]. The gut fungal mycobiome is related to tumorigenesis, and commensal fungi can be transformed into the pathogens by multiple environmental conditions [161].

Fungi are also ubiquitous on the skin, with two sets of fungi being involved in skin diseases: ascomycete dermatophytes, including the genera *Trichophyton*, *Microsporum*, and *Epidermophyton*, and basidiomycete fungi in the genus *Malassezia* [165,166]. Vaginal fungi typically belong to the *Candida* genus, *C. albicans* being most common [167]. There is clear evidence between symbiotic and pathogenic fungi and cancer development, mediated by the fungi’s ability to modulate various processes and pathways in cancer [14]. Recently published data suggest the “dietary nutrients-fungi-host” tripartite interaction is one of the most profound connections involved in tumor progression [168].

### 5.1. Pro-Cancer

The most common fungal genera and species generally associated with cancer are *Candida* (*C. albicans*, *C. glabrata*, and *C. tropicalis*), *Aspergillus* (*A. flavus* and *A. parasiticus*), and *Fusarium* (*F. verticilloides* and *F. proliferatum*) [14]. Thus, infections caused by both pathogenic fungi as well as opportunistic fungal infections with *Candida* sp. (*C. albicans*, *C. tropicalis*, *C. glabrata*, and *C parapsilosis*), *Aspegillus* sp., *Fusarium* sp., *Mucorales* sp., and *Rhizopus* are involved in skin cancer [14]. Most evidence supports the relationship between *C. albicans* and oral cancer development [169]. Thus, cancer progression may be promoted by *C. albicans* by carcinogenic metabolites production, inducing chronic inflammation, immune microenvironment remodeling, activation of pro-tumor pathways, and fungal–bacterial interaction [169].

Fungi are able to migrate from the gut lumen to different organs, such as the pancreas, where fungi have been detected in the TME of pancreatic ductal adenocarcinoma (PDAC) samples [170,171]. In humans and mouse models of PDAC, fungi increased about 3000-fold compared to health pancreatic tissue, and PDAC mycobiome differs from the gut or normal pancreas [171]. Dohlman et al. (2022) conducted a pan-cancer mycobiome analysis and found up to one fungal cell per 10^4^ human tumor cells [172]. *Malassezia* spp. was reported as the most prevalent fungus in PDAC in both mice and humans, so some antifungal medications protect mice against cancer progression [170]. *Blastomyces* was associated with tumor tissue in the lung [172]. *Candida* species, *Saccharomyces cerevisiae*, and *Cyberlindnera jadinii* are highly abundant in gastrointestinal (GI) tumor mycobime communities, whereas *Blastomyces* and *Malassezia* were also abundant in breast tumors [172]. Intestinal fungal dysbiosis with the enrichment of opportunistic fungi *Malassezia* and *Candida* was revealed in patients with HCC cancer compared with healthy subjects and cirrhosis patients [173]. Intratumoral fungi emphasize the ability to induce tumor growth through activation of the complement system [170].

The candidiasis-associated malignant development has been highlighted in many cancers, including breast cancer [174] and gastric cancer (GC) [175]. Current evidence suggests that the opportunistic *C. albicans* yeast increases the risk of carcinogenesis and metastasis through production of carcinogenic metabolites, inducing chronic inflammation and remodeling the immune microenvironment [169,176]. Thus, generation of carcinogenic by-products, such as nitrosamine, alcohol-derived carcinogenic agents, and heme oxygenase enzymes, induce inflammatory response, molecular mimicry, and epigenetic modifications, which encourage carcinogenesis and tumor progression [174]. Zhong et al. (2021) concluded that *C. albicans* can be considered as a fungal biomarker for GC in association with *Fusicolla acetilerea*, *Arcopilus aureus*, and *Fusicolla aquaeductuum* that were also increased, while *C. glabrata*, *Aspergillus montevidensis*, *Saitozyma podzolica*, and *Penicillium arenicola* were decreased in GC lesions [175].

Mycotoxins are fungal metabolites, such as aflatoxin B1 (AFB1), produced by *Aspergillus flavus* and *A. parasiticus* which grow in soil, nuts, and grains, known as natural carcinogenetic contaminants able to cause major mutagenic changes in the nucleotide sequence, leading to genomic instability and mutation [177,178]. AFB1 has the highest carcinogenicity among all mycotoxins [178]. Thus, exposure to aflatoxin is a potential risk factor for developing hepatocellular carcinoma [179] and breast cancer [180]. Exposure of MCF7 and MCF10A cells at low levels of AFB1 can induce modifications in the expression of key genes involved in cancer, cell cycle, apoptosis, and p53 pathways, as well as changes at the transcriptomic level of *BRCA1/2*, *p53*, *HER1/2*, *cMYC*, *BCL2*, *MCL1*, *CCND1*, *WNT3A*, *MAPK1/2*, *DAPK1*, and caspases 8/9 genes [180]. Additionally, AFB1 modulates ROS levels and increased cancer cell migration rates [180]. Fumonisins, including fumonisin B1 (FB1) and fumonisin B2 (FB2), a class of mycotoxins produced by *Fusarium* species, are considered probable human carcinogens due to their ability to induce OS and lipid peroxidation as well as DNA mutation and epigenetic alterations through disruption of sphingolipid metabolism that affects cellular signaling, membrane integrity, and apoptosis [181].

### 5.2. Anti-Cancer

Yeast can be used as a model organism for studying tumorigenic mechanisms, as well as for drug discovery, production of anti-cancer drugs at a large scale, and next-generation immunotherapy delivering [182]. Consequently, the use of yeast-based array technologies allows for the discovery of targetable genes for personalized cancer therapies [182]. Furthermore, the interaction between dietary nutrients, fungi, and host cells provides important insights into the application of fungi-targeted strategies in precision nutrition for cancer prevention and treatment [168].

Beta-lactam antibiotics, such as penicillin, produced by *Penicillium chrysogenum/rubens*, and cephalosporin antibiotics, can be isolated from fungi. Penicillin is an old and highly used antibiotic able to disrupt mitochondrial function and energy metabolism in colon cancer cells, leading to autophagic apoptosis and inhibition of cancer cell growth and metastasis [183]. Cephalosporins are also a large group of antibiotics derived from a metabolite of the mold *Cephalosporium coronarium*, which are able to increase the effects of radiotherapy by increasing oxidative DNA, proteins, and membrane lipids damage, possible through ROS overgeneration, leading to toxic effects on cancer cells [184]. He et al. (2021) showed that cephalosporin antibiotics selectively and specifically target nasopharyngeal carcinoma cells via heme oxygenase 1 (HMOX1)-induced ferroptosis [184].

Cancer cell apoptosis can be induced by exposure to aflatoxin B1 (ATB1), known as one of the most carcinogenic aflatoxins, in the 4T1 mouse mammary invasive carcinoma cell line [185]. Arenicolins A, C-glycosylated depsides from *Penicillium arenicola*, exhibited cytotoxicity toward colorectal carcinoma (HCT-116), neuroblastoma (IMR-32), and ductal carcinoma of the breast (BT—474) mammalian cell lines [186]. Apoptosis and anti-proliferative effects can be also induced by the heat-killed form of the common probiotic *Saccharomyces cerevisiae* baker’s yeast through increasing PTEN expression in CRC cells [187]. Moreover, it was demonstrated that *S. cerevisiae* exerts anti-skin cancer activity through downregulation of BCL2 expression and BAX upregulation, and increasing the expression of caspases 3/8/9 [188]. Recombinant *S. cerevisiae* can be used as a non-pathogenic and non-toxic vaccine vehicle to induce immune responses to foreign tumor-associated antigens, so vaccination with this heat-killed recombinant yeast expressing the human carcinoembryonic antigen (CEA) induces CEA-specific immune responses, reduces tumor burden, and leads to increasing overall survival in CEA-transgenic mice [189]. Yeasts can be also used as protection and delivery vehicles. A combination of probiotic yeasts and bioactive compounds has been effective in treating different types of cancer [190]. Thus, the heat-killed form of *Saccharomyces cerevisiae* in combination with nanocarriers, such as curcumin-loaded niosomal nanoparticles (NPs), has been used as an effective anti-cancer agent against the CRC cell line [190]. This nanocombination led to the downregulation of genes involved in CRC metastasis, including matrix metalloproteinases 2/9 (MMP2/9) and collagen COL10A1, that results in apoptosis and cell cycle arrest in cancer cells [190].

Endophytic fungi residing in the tissues of medicinal plants and their bioactive fungal metabolites are a promising source for the development of anti-cancer agents for BC drug development and therapy [191]. In addition, endophytic fungal symbionts from marine flora and fauna produce secondary metabolites with anti-cancer properties [192]. Thus, averufin isolated from the marine sponge-derived endophytic fungus *Penicillium verruculosum* has good pharmacokinetic properties against myeloid leukemia [192]. The *Cladosporium* sp. UR3 strain, isolated and purified from the Red Sea sponge, *Hyrtios* sp., contains a plethora of metabolites with AKT1, ESR1, and EGFR tyrosine kinase inhibitory potential and a powerful activity against colorectal (CACO2), BC (MCF7), and HCC (HEPG2) cell lines [193]. Several bioactive compounds, including altertoxin X isolated from *Cladosporium* species, revealed anti-cancer activity and could serve as new therapeutic agents for BC treatment, using the estrogen receptor alpha transcription factor as drug target [194]. Moreover, fusarubin (FUS) and anhydrofusarubin (AFU) isolated from *Cladosporium* species inhibit cell proliferation and increase apoptosis in human acute myeloid leukemia and other hematologic cancer cell lines [195]. At a high concentration, FUS upregulated p21 expression and stability in a p53-dependent manner, decreasing ERK and AKT phosphorylation [195]. Pro-cancer and anti-cancer effects of fungal infection are summarized in Table 3.

## 6. Protozoa

Numerous species of protozoa, including *Toxoplasma gondii* [196,197,198], *Plasmodium* [199], *Trypanosoma cruzi* [200], *Trichomonas vaginalis* [201], *Giardia duodenalis* [202], *Entamoeba histolytica* [203], *Leishmania* [204], *Neospora caninum* [205], as well as protozoan-derived molecules, may have pro-tumorigenic or anti-neoplastic potential, so parasites emphasize great interest in cancer therapy, resulting in pathogen-based cancer therapies [15,206,207].

### 6.1. Pro-Cancer

Parasite protozoa induce modification in cell cycle, metabolism, glycosylation, DNA mutations, apoptosis, cell senescence, metastatic cascade, and angiogenesis [200]. Even if it is found everywhere in the world, in developing countries, millions of people yearly die due to amoebiasis, an infection caused by *Entamoeba histolytica*, a pathogen that raises the incidence of colorectal cancer (CRC), probably by inducing microsatellite instability [203,208]. *Toxoplasma gondii* is an obligate neurotropic intracellular parasite that causes toxoplasmosis that is estimated to infect nearly one-third of the world human population [209]. In the USA and UK, 8–22% of the people are infected with *T. gondii*, while in Europe, South America, and Central Africa, estimates ranged between 30 and 90% [210]. *T. gondii* regulates many signaling pathways, such as energetic metabolism, immune response, and inflammation, so the parasite is involved in carcinogenesis and cancer development [197]. Evidence suggests that this parasite may stimulate the development of solid tumors while inhibiting hematologic cancers [198]. Thus, in many cancers, *T. gondii* infection can modulate host immunosuppression to restrain tumor growth by upregulating the expression of interleukin-12 (IL-12) and interferon gamma (IFN-γ) [211]. *Trichomonas vaginalis* is a prevalent extracellular eukaryotic parasite that causes trichomoniasis, known as the most widespread sexually transmitted infection worldwide; thus, *T. vaginalis* is most prevalent among women and Black people, with an estimated prevalence of 5.3% in women and 0.6% in men, and an incidence of 156 million cases worldwide [212]. *T. vaginalis* was associated with a higher risk of cervical cancer, especially when co-infected with HPV [213,214]. It was also suggested that men with trichomoniasis had a higher risk of developing prostate cancer [214]. Leishmaniasis is a parasitic disease of tropics and subtropics caused by *Leishmania* species; 12 million people in over 90 countries are affected by leishmaniasis, with an annual incidence of 0.9–1.6 million, between 20,000 and 30,000 deaths/year, and 350 million people at risk to become infected [215]. *Leishmania sp*. infection seems to play a significant role in the pathogenesis and prognosis of several cancers, producing chronic inflammation, metabolic oxidative stress, apoptosis inhibition, and inhibition of tumor suppressors [204].

### 6.2. Anti-Cancer

The host immune system becomes activated and enhanced by protozoan parasites and their derived products, thereby inhibiting tumor growth, angiogenesis, and metastasis [216]. In a mouse model of CRC, exosomes derived from dendritic cells infected with *Toxoplasma gondii* showed anti-cancer activity, mainly based on reducing the proportion of immunosuppressive myeloid-derived suppressor cells (MDSCs) and by inhibition of STAT3 signaling in these cells, leading to tumor growth inhibition [206]. Wei et al. (2018) showed that *T. gondii* dense granule protein 15 (GRA15) induces apoptosis in choriocarcinoma JEG-3 cells by induction of endoplasmic reticulum stress [217]. Moreover, *T. gondii* tachyzoites have shown anti-cancer activity in BC mouse models based on MCF7 and MDA-MB-231 cell lines due to the ability to regulate several signaling pathways at the transcriptome level by altering BRCA1, MYC, and IL-6, which are known to inhibit mammary tumor growth and migration [218]. Chen et al. (2021) showed that *Plasmodium*, an intracellular parasite, also activates the immune system of the host, counteracting the tumor immunosuppressive microenvironment, inhibiting angiogenesis, tumor growth, and metastasis, increasing survival time in murine cancer models [199]. *Plasmodium (P. falciparum and P. vivax)* is the most common parasitic agent in humans and animals [219]. Infection with malaria parasites has an anti-tumor effect, stimulating innate and adaptive immunity in murine HCC models, so *Plasmodium* was proposed as an HCC vaccine vector [219].

*Trypanosoma cruzi*, the etiological agent of Chagas disease or trypanosomiasis, emphasizes both a pro-tumoral role as well as anti-tumor effects by toxins that destroy neoplastic cells, modulate the energetic metabolism of infected cells, and stimulate the immune system through lysates and infection [200]. In the Americas, six millions persons are estimated to have Chagas disease [220], with a global prevalence around 11.3% [221]. Macrophages rapidly engulfed tumor cells, such as the canine mammary carcinoma cell line and canine transmissible venereal tumor (CTVT) cells, when treated with recombinant calreticulin (rTcCRT), an endoplasmic reticulum-resident chaperone translocated/externalized form *T. cruzi*, demonstrating that rTcCRT is able to boost the host immune responses [222]. Thus, there are reports which indicate that in patients with Chagas’s disease, cancer is a really rare disease, due to rTcCRT, which emphasizes antiangiogenic and anti-tumor activities, mainly based on the capacity of rTcCRT to inhibit endothelial cell proliferation in mammary tumors [223].

The coccidian parasite *Neospora caninum* (Apicomplexa: Sarcocystidae), non-pathogenic in humans but phylogenetically close to *Toxoplasma gondii* [224], also emerged as an oncolytic protozoan in human oncology due to its ability to strongly inhibit or even eradicate tumor development when injected in a B16F10 murine melanoma model [225]. *N. caninum* had the ability to destroy infected malignant cells, reactivate the immunosuppressed immune cells, and generate an anti-tumor response dependent of natural killer cells and CD8^+^ T-cells, in association with IFN-γ secretion in the TME [205].

A study conducted by Vicier et al. (2019) sustained that high level of serum cytokines and *T. vaginalis* seropositivity at diagnosis were not associated with high-grade lethal prostate cancer [226]. It was also reported that leishmanial sphingolipid-1 (LSPL1) from *Leishmania donovani* has anti-neoplastic potential through cellular growth modulation, apoptosis induction, and angiogenesis silencing in sarcoma 180 cell-associated cancer, also exerting anti-neoplastic effects in B16F10 melanoma cells by regulation of angiogenesis and inflammatory response [227,228]. *Leishmania* parasites and cancer cells share several upregulated or downregulated common proteins related to survival, development, pathogenicity, and metabolic pathways, so several opportunities for therapeutic targeting in both leishmaniasis and cancer could be possible [229]. Among these common proteins between leishmaniasis and cancer, Rashidi et al. (2021) cited protein disulfide-isomerases (PDIs), which affect survival progression, and metastasis in lymphoma, brain, ovarian, kidney cancers, and others, as well as the pathogenicity and survival of parasite, superoxide dismutases (SODs) with the protective function in *Leismania* parasites, influencing apoptosis in brain tumors or progression and metastasis in pancreatic cancer cells, phospholipase, tyrosyl-DNA-phosphodiesterase-1 (TDP1), HSP60, HSP90, aldehyde dehydrogenase (ALDH), topoisomerases (TOPs), proliferating cell nuclear antigen (PCNA), tubulins, voltage-dependent anion-selective channel protein 1 (VDAC1), mitochondrial import receptor subunit (TOM40), ornithine aminotransferase (OAT), selenoproteins and selenoamino acid, and phosphoglycerate kinase-1 (PGK1) [229]. Pro-cancer and anti-cancer effects of protozoa parasite infection are synthetized in Table 4.

## 7. Microalgae

Hypoxia is an important predictor for poor clinical outcome and treatment resistance in solid tumors [231]. The chloroplasts present in algae are a valuable oxygen source through photosynthesis, alleviating hypoxia in TME and allowing for tumor growth inhibition [18]. Chlorophyll (Chl) from microalgae also produces ROS during laser irradiation, enhancing tumor apoptosis [34]. Algae-inspired microrobots (AIMs) are recommended to fight against cancer due to their biocompatibility, autofluorescence, photothermal convertibility, stimuli-responsiveness, generation of photodynamical radicals, and pharmaceutical activity [46,232].

Both photodynamic therapy (PDT), as well as sonodynamic therapy (SDT), are used as non-invasive or minimally invasive alternatives to chemotherapy and radiotherapy to inhibit the spread of malignant tumor cells. PDT combines a photosensitizer (PS) such as Chl, tissue molecular oxygen, and a source of light energy for PS photoactivation [233]. Chlorophyll-loaded mesoporous silica nanoparticle (Chl-MSNs) platforms under red and blue laser irradiation showed a weak toxic effect in the destruction of the hepatocellular carcinoma cell line (HepG2), highly aggressive MDA-MB-231 BC cell line, and human skin fibroblasts (HSF) [233]. Moreover, Chl produces anti-proliferative effects in pancreatic cancer cell lines (PaTu-8902, MiaPaCa-2, and BxPC-3) [234]. Motile algae have been used as biomotors of biohybrid microrobots, such as the algae-NP(DOX)-robots, that were designed for localized delivery of drug-loaded NPs against melanoma lung metastasis [235]. This type of robot combines green microalgae, emphasizing autonomous and continuous propulsion with red blood cell (RBC) membrane-coated biodegradable polymeric poly(lactic-co-glycolic acid) (PLGA) NPs containing doxorubicin (DOX), a wide-used chemotherapeutic agent [235]. Intratracheal administration of algae-NP(dox) robots resulted in a more effective accumulation of the loaded drugs into the lung due to the slow uptake of robots by alveolar macrophages [235]. To improve the efficiency of immunotherapy, another photosynthetic microrobot has been designed, combining a natural freshwater photosynthetic/green microalgae, *Chlorella vulgaris*, and an engineered dendritic cell (DC) membrane, overexpressing tumor necrosis factor (TNF) ligand proteins OX40L, 4-1BBL, and CD70 [236]. This robot enhances the activity and proliferation of the effector and memory T cells, and when combined with programmed death-1 (PD-1) antibody, the robot prevents tumor relapse and metastasis in a mouse model [236]. *C. vulgaris* was also used as a carrier for doxorubicin (CV@DOX) for osteosarcoma therapy [237]. CV@DOX releases DOX in the acidic TME and generates oxygen under laser irradiation [237]. Surface-engineered microalgae *C. vulgaris* modified with metallic-organic framework nanoparticles (Chl-MOF) are successfully designed for synergistic photo-sonodynamic therapy and immunotherapy in breast cancer treatment [238]. Thus, Chl-MOF bioaccumulation in tumor site is higher to alleviate tumor hypoxia, produce ROS during laser and ultrasound (US) irradiation, enhance tumor cell apoptosis, increase the effects of synergistic PDT and SDT, increase natural killer cell cytotoxic activity, increase dendritic cell antigen-presenting ability, reverse the characteristics of the immunosuppressive TME, and induce an important anti-tumor response [238].

## 8. Limitations and Future Challenges

Mutations, genetic instability and individual genetic resistance, epigenetic factors, enhanced drug efflux, inhibition of apoptosis, and hypoxia are several causes responsible for development of resistance against conventional anti-cancer treatments. To overcome this great concern in clinical practice, reduce tumor burden and secondary undesirable side effects of existing therapies, and improve their efficacy as well as the quality of patients’ lives, conventional treatments are more and more used in a combinatorial mode with modern approaches based on small biological fighters against cancer, such as viruses, bacteria, archaea, fungi, protozoa, and microalgae. Thus, the interaction between tumor cells, immune cells, and microbiota is considered as an immuno-oncology-microbiome (IOM) axis [239]. Microbiome-based diagnosis of cancer is a complementary supplement for genomics- and proteomics-based approaches [240]. In this context, artificial intelligence-enabled microbiome-based diagnosis models for a large spectrum of cancer types indicated that certain sets of microbes reveal the differences among cancer patients and healthy people [240].

Oncolytic virotherapy, based on several viruses with their advantages and disadvantages, is a strategy based on selective virus infection and replication in cancer cells [241]. Many viral genes/proteins are able to reprogram host cellular pathways involved in proliferation, differentiation, apoptosis and other types of cell death, genomic integrity and mutation, or immune surveillance. Thus, oncoviruses integrate their genes into the host genome, activating oncogenes and/or promoting oncogenic proteins, thus inducing malignant transformation and cancer development through disruption of cell cycle regulation, apoptosis, and DNA damage repair (DDR) mechanisms, leading to uncontrolled cell proliferation. On the opposite side, a plethora of oncolytic or attenuated and/or genetically engineered viruses are used in anti-cancer virotherapy due to their oncolytic effect on cancer cells or ability to target specific tumor areas, such as inaccessible hypoxic regions. Moreover, viral-specific enzymes are able to convert, together with a cancer cell’s own enzymatic machinery, the non-toxic form of a chemotherapeutic drug into the cytotoxic form, leading to lysis of transgene-expressing cells and those surrounding them. In addition, commensal viruses can play an important role in cancer suppression. Oncolytic viruses can be used to genetically modify a wide range of cancer cells and trigger the expression of tumor-specific antigens, or kill tumor cells, releasing soluble antigens and interferons and, finally, boosting the anti-cancer immune responses. Plant viruses and plant virus nanoparticles (PVNPs) can be engineered for targeted therapeutic agent delivery, imaging applications, and immunotherapy. Identification of oncogenic DNA and RNA viruses, understanding their molecular mechanisms of action leading to tumorigenesis and cancer development, as well as discovery of new approaches for treatment and/or prevention of viral infections known to lead to cancer are crucial. Furthermore, identification of combinatorial therapies that include new generations of oncolytic or engineered viruses able to stimulate optimally the host immunity and kill cancer stem cells to improve efficacy of anti-tumor treatments in metastatic cancer is still a challenge in oncolytic virotherapy.

As well as viruses, bacteria are important components of human microbiomes. Bacteria are also recognized as opportunistic tumor inhabitants, producing environmental stress that alters the TME. However, only several species of bacteria are recognized as oncobacteria directly related with carcinogenesis. The good side of bacteria resides from genetically modified bacteria, which are designed to express reporter genes, cytotoxic proteins or anti-cancer agents, and tumor-specific antigens, triggering a specific immune response in patients, whereas bacterial toxins and enzymes can selectively cause apoptosis and cell cycle inhibition, and could be combined with anti-tumor drugs or radiation therapy to enhance the efficacy or to alleviate the side effects of conventional anti-cancer therapies. Anaerobic bacteria can be incorporated into bacteria-based biohybrid platforms able to colonize the hypoxic regions in tumors, where they deliver chemotherapeutic drugs that selectively accumulate only to the tumor site. Bacteria can also fight against cancer cells by depletion of nutrients and oxygen required for cancer cell metabolism. Image-guided and thermally or magnetically controlled bacteria could serve as imaging agents and delivery platforms for anti-tumor treatment. Archaea act as a double-edged sword, secreting different bioactive compounds that can inhibit cancer cell proliferation, or producing oncogenic metabolites that can regulate the TME and promote carcinogenesis by microbiota modulation, aberrant signaling pathways, ROS production, DNA damage, inflammation, genotoxicity, tumor cell proliferation, and cell differentiation. Archaeosomes synthetized from a membrane’s natural lipids extracted from archaea or synthetic archaeal lipids can transport and deliver anti-cancer-loaded drugs, proteins, peptides, genes, antioxidants, and cells to the tumor site. The toxicity of bacteria and their genetic instability and mutations are recognized as the most important problems in bacteriotherapy [242]. Furthermore, compared with conventional probiotics, next-generation probiotics represent an innovative group of beneficial bacterial strains able to target tumor cells for selective and specific drug delivery, produce bioactive compounds, successfully interact with commensal and pathogenic bacteria and fungi, modulate the host immunity, and participate in combinatorial therapies for personalized probiotic therapies able to reduce tumor burden and enhance the success of conventional anti-cancer therapies [243].

Pathogenic fungi and intratumoral mycobiota can induce tumor growth, but there are also important advantages of commensal fungi from human microbiomes, fungal endophytes that colonize plant tissues, and their bioactive compounds that show great anti-malignant potential. Interestingly and challengingly, the gut commensal fungi can be transformed into the pathogens by multiple environmental conditions [161]. Understanding the fungi mechanisms to fight against cancer is essential for the development of novel opportunities in cancer prevention and treatment. Advances in metagenomics and accumulation of multi-omics data allowed for understanding the structure of the mycobiome and its contribution to tumorigenesis and cancer development. Dietary nutrients-fungi-host interaction is considered today as one of the most profound connections involved in tumor progression [168]. However, the great variability of mycobiomes in terms of geographic distribution, prevalence, diet and nutritional habits, ethnicity, cancer type and subtype, and other factors, mechanisms of the carcinogenic effects of fungi-derived metabolites, as well as the complex bacteria–fungi relationships, are not fully understood. The diversity of software tools and the complexity of analytical methods still present a challenge for detecting, identifying, and analyzing the holistic and diverse intratumoral mycobiomes [239]. In the future, the need for standardized protocols and computational methods for taxonomic identification at higher levels of fungal strains in order to understand the host–mycobiome interactions and to clarify the molecular mechanisms of mycobiome in tumorigenesis is crucial [239].

**Figure 1:**
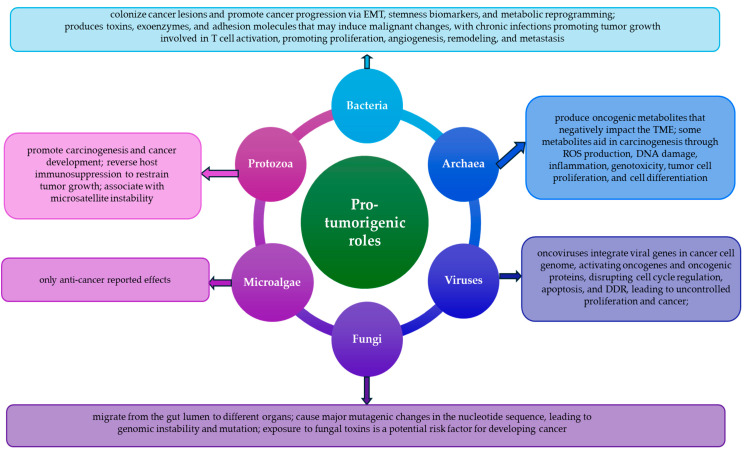
Pro-tumorigenic roles of small biological entities. Abbreviations: EMT—epithelial-mesenchymal transition; DDR—DNA damage response.

**Figure 2:**
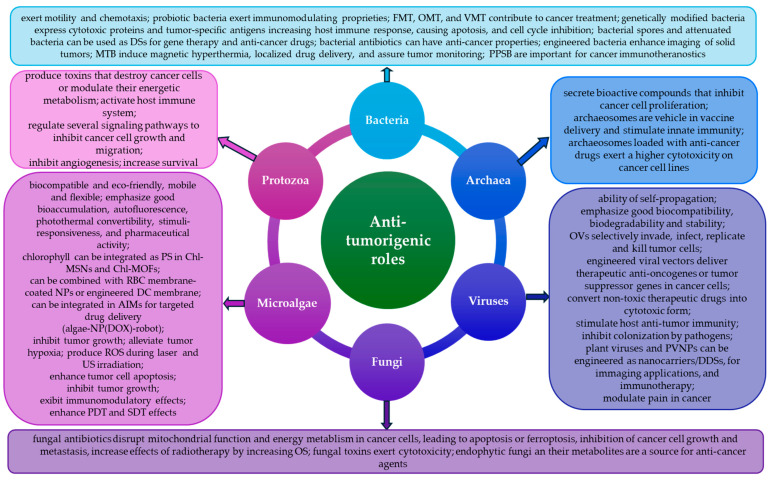
Anti-tumorigenic roles of small biological fighters against cancer. Abbreviations: AIMs—algae-inspired microrobots; Chl-MOF—surface-engineered *C. vulgaris* modified with metal-organic framework NPs; Chl-MSNs—chlorophyll-loaded mesoporous silica nanoparticles; *C. vulgaris*—*Chlorella vulgaris*; DC—dendritic cell; DDSs—drug delivery systems; DSs—delivery systems; DOX—doxorubicin anti-cancer drug; FMT—fecal microbiota transplantation; MTB—magnetotactic bacteria; NPs—nanoparticles; OMT—oral microbiota transplantation; OS—oxidative stress; OVs—oncolytic viruses; PPSB—purple photosynthetic bacteria; PS—photosensitizer; PVNPs—plant virus nanoparticles; RBC—red blood cell; PDTD—photodynamic therapy; ROS—reactive oxygen species; SDT—sonodynamic therapy; US—ultrasounds; and VMT-vaginal microbiota transplantation.

Numerous species of protozoa as well as protozoan-derived molecules may have pro-tumorigenic or anti-neoplastic potential, so these parasites emphasize great interest in cancer therapy, resulting in pathogen-based cancer therapies. Parasites induce modification in the cell cycle, metabolism, glycosylation, DNA mutations, apoptosis, cell senescence, metastatic cascade, and angiogenesis, but on the other side, the host immune system becomes activated and enhanced by protozoa parasites and their derived products, thereby inhibiting tumor growth, angiogenesis, and metastasis. The discovery of numerous species of protozoa and their components with anti-tumor potential introduced a novel direction for the development of protozoa-based anti-cancer treatment that necessitates further extensive studies [15]. Microalgae-inspired microrobots are also recommended to fight against cancer due to their biocompatibility, autofluorescence, photothermal convertibility, stimuli-responsiveness, generation of photodynamical radicals, and pharmaceutical activity. Fortunately, there is no limit to the development of novel approaches in cancer biomedicine. This review summarized the most important pro-cancer (Figure 1) as well as anti-cancer (Figure 2) roles of viruses, bacteria, archaea, fungi, protozoa, and microalgae.

## 9. Conclusions

Despite the progress made in oncological theranostics, cancer remains a global health challenge and a leading cause of death worldwide. Conventional tumor treatment protocols, including surgery, chemotherapy, radiation therapy, targeted therapy, and immunotherapy, are not beneficial for all patients. In this review, we emphasized how small biological entities, such as viruses, bacteria, archaea, fungi, protozoans, and microalgae, as well as their related structural compounds and toxins/metabolites/bioactive molecules, can prevent and suppress cancer, or conversely, regulate malignant initiation, progression, metastasis, and responses to different therapies. In conclusion, nanotheranostics that employ biomimetic approaches based on biology-inspired strategies could maximize cancer diagnostic and therapy efficiency, leading to improved patients’ quality of life.

## Figures and Tables

**Table 1:** Anti-cancer roles of several human and plant engineered viruses.

Viruses	Anti-Cancer Effects
Engineered adenoviruses (OAd)	deliver transgenes specifically to cancer cells while sparing normal cells, making them take up radioactive iodine, which can be exploited for noninvasive imaging and radiotherapy [92]; become promising options for invasive BC [92]; target paclitaxel resistant ER+ BCSCs [92] and PDAC cells [93]
Engineered adeno-associated viruses (AAVs)	used as gene delivery vectors, selectively target BC cells [67]
Genetically engineered Herpes simplex virus (oHSV)	selectively targets cancer cells while sparing normal cells, kills cancer cells mainly by boosting host innate or/and adaptive immunity [94]
Recombinant mumps virus	acts as a promising oncolytic agent, emphasizing anti-cancer activity against various cancers, including advanced gynecological cancer [95]
Engineered Variola virus	oncolytic agent, selectively kills cancer cells, delivery vehicle for anti-cancer transgenes as well as a vaccine carrier for tumor-associated antigens and immunoregulatory molecules in cancer immunotherapy [91]
Human endogenous retroviruses (HERs)	play divergent roles in BC carcinogenesis [96]
Tobacco mosaic virus (TMV) NPs	component of delivery platforms for pheanthriplatin, which can be released in acidic TME [103]; loaded with MTO, act on cancer cell lines and mouse model of TNBC [104]
Potato virus X (PVX) NPs	emphasize good tumor penetration [101]; PVX-NPs-DOX showed efficacy on OC, BC, and cervical cancer cell lines, reducing tumor growth [101]
Cowpea mosaic virus (CPMV) NPs	strong immunostimulatory properties, reshape the immunosuppressive TME in murine orthotopic ovarian cancer model by modulating cytokine secretion [106]

Abbreviations: BC—breast cancer; BCSCs—breast cancer stem cells; DDSs—drug delivery systems; MTO—mitoxantrone; OC—ovarian cancer; PDAC—pancreatic ductal adenocarcinoma; PVX-NPs-DOX—delivery system based on potato virus X nanoparticles loaded with doxorubicin, and TME—tumor microenvironment.

**Table 2:** Anti-cancer activities of bacterial strains and their applications in bacteriotherapy.

Bacteria	Anti-Cancer Effects
*Lactobacillus* spp. and *Bifidobacterium bifidum* strains (probiotics)	involved in cell cycle regulation, inhibit cell proliferation and activate pro-caspases and BAX, downregulate BCL-2 [120]
*Bifidobacterium longum*	engineered, targets tumor hypoxia, enhances the imaging of solid tumors and improves the efficiency of HIFU treatment by enhancing NP targeting ability and increasing their retention time and effects of engineered HIFU synergists [131]
*Salmonella typhimurium*	aggregates and proliferates inside TME, stimulating inflammation and promoting anti-tumor immunity [124,125]; modified or less toxic/attenuated are used as DDSs, inhibiting tumor growth and metastasis or promoting apoptosis in ovarian murine tumors [124], delivers toxins that induce apoptosis in cancer cells [126]
*Clostridium perfringens*	produces enterotoxin, induces disruption of membrane permeability, influx of calcium ions, and cancer cell death [128]
*Streptomyces peucetius* var. *caesius*	produces DNR and DOX used for anti-cancer activities in acute leukemia and solid tumors, triggers both DNA double-strand breaks and histone eviction [130]
*Escherichia coli* (non-pathogenic)	engineered, selectively targets, colonizes, and proliferates within solid tumors, especially in hypoxic regions [132]; component of DDSs [133]
MTB strains	synthesize and contain magnetosomes for targeted therapy, enhance the effects of PTT [45,135]
PPSB strains	immunotheranostics [134]

Abbreviations: DDSs—drug delivery systems; DNR—daunorubicin; DOX—doxorubicin; HIFU—high-intensity focused ultrasound; MTB—magnetotactic bacteria; PPSB—purple photosynthetic bacteria; PTT—photothermal therapy; and TME—tumor microenvironment.

**Table 3:** Pro-cancer and anti-cancer roles of fungal infection.

Fungal Infections	Associated with	Pro-Cancer Effects	Anti-Cancer Effects
*Candida albicans*/ candidiasis	human gut opportunistic inhabitant, oral cancer [169], skin cancers [14], GI tumors (GC), HCC [173], BC [174]	carcinogenic metabolites production, chronic inflammation, immune environment remodeling, activation of tumor-pathways, and fungal–bacterial interaction	-
*Malassezia* spp.	human gut opportunistic inhabitant, PDAC [170], BC [172], GI tumors, HCC [173]		-
*Aspergillus flavus* *A. parasiticus*, *Fusarium* spp.	potential risk for HCC [179] and BC [180]	produce exogenous toxins (aflatoxins, fumonisins) cause DNA mutations and genomic instability [177,178]; induce OS, protein and lipid peroxidation, epigenetic modifications, affect cellular signaling, membrane integrity, and apoptosis	aflatoxins can also induce cancer cells apoptosis in 4T1 mouse mammary invasive carcinoma cell line [185]
*Penicillium chrisogenum* *P. rubens*	many cancers	-	produce penicillin that disrupts mitochondrial function and energy metabolism in colon cancer cells, leading to autophagic apoptosis and inhibition of cancer cell growth and metastasis [183]
*Penicillium arenicola* (endophytic fungi)	CRC, neuroblastoma, BC [186]	-	produces arenicolins that exhibit cytotoxicity on cancer cell lines [186]
*Penicillium verruculosum* (endophytic fungi)	myeloid leukemia [192]	-	averufin has good pharmacokinetic properties [192]
*Cephalosporium* spp.	many cancers	-	produces cephalosporins that increase the effects of radiotherapy by increasing DNA, proteins and membrane lipids oxidative damage, possible through ROS overproduction, leading to toxic effects on cancer cells, selectively and specifically target nasopharyngeal carcinoma cells via HMOX1-induced ferroptosis [184]
*Cladosporium* spp. (endophytic fungi)	CRC, BC, HCC cell lines [193]	-	contains metabolites with AKT1, ESR1, and EGFR tyrosine kinase inhibitory potential and powerful activity against cancer cell lines [193]; FUS and AFU inhibit cancer cell proliferation and increase apoptosis in human acute myeloid leukemia and other hematologic cancer cell lines, FUS upregulates p21 expression and stability in a p53-dependent manner, decreasing ERK and AKT phosphorylation [195]
*Saccharomyces cerevisiae* (Brewer’s yeast)	human gut opportunistic inhabitant, CRC [187], skin cancer [188]		heat-killed form induces apoptosis and reduces cancer cell proliferation, PTEN overexpression [187]; BCL2 downregulation, BAX and caspases 3/8/9 upregulation [188]; vaccination reduces tumor burden and increases survival in CEA-transgenic mice [189]; in different nano-combinations, downregulates genes involved in CRC metastasis, including MMP2/9 and COL10A1, inducing apoptosis and cancer cell cycle arrest [190]

Abbreviations: AFU—anhydrofusarubin; AKT1—protein kinase B; BC—breast cancer; BCL2—B-cell lymphoma 2; CEA—carcinoembryonic antigen; COL10A1—collagen type X A1; CRC—colorectal cancer; EGFR—epidermal growth factor receptor; ERK—extracellular signal-related kinase; ESR1—estrogen receptor 1; FUS—fusarubin; GC—gastric cancer; GI—gastrointestinal tract; HCC—hepatocellular carcinoma; HMOX1—heme oxygenase 1; MMP2/9—matrix metalloproteinases 2/9; OS—oxidative stress; PDAC—pancreatic ductal adenocarcinoma; and ROS—reactive oxygen species.

**Table 4:** Pro-tumorigenic and anti-cancer roles of protozoa parasites infection.

Protozoa/Infection	Associated with	Pro-Cancer Effects	Anti-Cancer Effects
	many cancers	modification in cell cycle, metabolism, glycosylation, DNA mutations, apoptosis, cell senescence, metastatic cascade, angiogenesis [200]	activation of host immune system, inhibition of tumor growth, angiogenesis, and metastasis [216]
*Entamoeba histolytica*/ amoebiasis	CRC [203]	microsatellite instability [203]	-
*Toxoplasma gondii*/ toxoplasmosis	solid tumors (anti-cancer activity on MCF7 and MDA-MB-231 BC cells [218])	regulates many signaling pathways (energetic metabolism, immune response and inflammation), involved in carcinogenesis and cancer development [198]	inhibits hematologic cancers, reverses host immunosuppression to restrain tumor growth, upregulating IL-12 and IFN-γ [211]; exosomes of infected DCs showed anti-cancer activity, by inhibition of STAT3 signaling in MDSCs, leading to tumor growth inhibition [206]
*Trichomonas vaginalis*/ trichomoniasis	cervical and prostate cancer [213,214]	causes trichomoniasis co-infected with HPV [213,214]	however, high level of serum cytokines and *T. vaginalis* seropositivity at diagnosis were not associated with high-grade lethal prostate cancer [226]
*Leishmania* spp./ leishmaniasis	several cancers (skin, lymphoma, HCC) have been diagnosed in patients with a history of leishmaniasis [204,230]	chronic inflammation, epigenetic alterations (DNA methylation in macrophages), metabolic and OS, apoptosis inhibition, inhibition of tumor suppressors, tumorigenesis [204,230]	LSPL1 from *Leishmania donovani* has anti-neoplastic potential through cellular growth modulation, apoptosis induction and angiogenesis silencing in sarcoma 180 cell-associated cancer, also exerting anti-neoplastic effects in B16F10 melanoma cells by regulation of angiogenesis and inflammatory response [227,228]
*Plasmodium* spp. (*P. falciparum*, *P. vivax*)/ malaria	HCC [219]	-	vector vaccine for HCC immunotherapy [219]; activates the immune system of the host (induces IFN-γ and TNF-α and activated immune cells NK and DCs), inhibits angiogenesis, tumor growth, and metastasis, increases survival time mice models [199]
*Trypanosoma cruzi* /trypanosomiasis/Chagas disease	in patients with Chagas’s disease, cancer is a really rare disease [200]	toxins can have a pro-tumoral role [200]	stimulates the immune system through lysates and infection, produces toxins that kill cancer cells or modulates cellular energetic metabolism [200]
*Neospora caninum*	B16F10 murine melanoma model [225]	-	oncolytic protozoan in human oncology, strongly inhibits or even eradicates tumor development; destroys infected malignant cells, reactivates immune cells, and generates an anti-tumor response dependent of NK cells and CD8^+^ T-cells, in association with IFN-γ secretion in TME [205]

Abbreviations: BC—breast cancer; CRC—colorectal cancer; DCs—dendritic cells; HCC—hepatocellular carcinoma; HPV—human papilloma virus; IFN-γ—interferon gamma; LSPL1—leishmanial sphingolipid-1; MDSCs—myeloid-derived suppressor cells; NK—natural killer; and TME—tumor microenvironment.

## Data Availability

Not applicable.
